# Neurobehavioral impairments predict specific cerebral damage in rat model of subarachnoid hemorrhage

**DOI:** 10.21203/rs.3.rs-2943917/v1

**Published:** 2023-05-19

**Authors:** Daniel G Lynch, Kevin A Shah, Keren Powell, Steven Wadolowski, Willians Tambo Ayol, Joshua J Strohl, Prashin Unadkat, David Eidelberg, Patricio T Huerta, Chunyan Li

**Affiliations:** Donald & Barbara Zucker School of Medicine at Hofstra/Northwell; North Shore University Hospital; The Feinstein Institutes for Medical Research; The Feinstein Institutes for Medical Research; Elmezzi Graduate School of Molecular Medicine; The Feinstein Institutes for Medical Research; Elmezzi Graduate School of Molecular Medicine; The Feinstein Institutes for Medical Research; The Feinstein Institutes for Medical Research; The Feinstein Institutes for Medical Research

**Keywords:** Subarachnoid hemorrhage, neurobehavioral dysfunction, regional brain damage, delayed cerebral ischemia, early brain injury

## Abstract

Subarachnoid hemorrhage (SAH) is a severe form of stroke that can cause unpredictable and diffuse cerebral damage, which is difficult to detect until it becomes irreversible. Therefore, there is a need for a reliable method to identify dysfunctional regions and initiate treatment before permanent damage occurs. Neurobehavioral assessments have been suggested as a possible tool to detect and approximately localize dysfunctional cerebral regions. In this study, we hypothesized that a neurobehavioral assessment battery could be a sensitive and specific early warning for damage in discrete cerebral regions following SAH. To test this hypothesis, a behavioral battery was employed at multiple time points after SAH induced via an endovascular perforation, and brain damage was confirmed via postmortem histopathological analysis. Our results demonstrate that impairment of sensorimotor function accurately predict damage in the cerebral cortex (AUC: 0.905; sensitivity: 81.8%; specificity: 90.9%) and striatum (AUC: 0.913; sensitivity: 90.1%; specificity: 100%), while impaired novel object recognition is a more accurate indicator of damage to the hippocampus (AUC: 0.902; sensitivity: 74.1%; specificity: 83.3%) than impaired reference memory (AUC: 0.746; sensitivity: 72.2%; specificity: 58.0%). Tests for anxiety-like and depression-like behaviors predict damage to the amygdala (AUC: 0.900; sensitivity: 77.0%; specificity: 81.7%) and thalamus (AUC: 0.963; sensitivity: 86.3%; specificity: 87.8%), respectively. This study suggests that recurring behavioral testing can accurately predict damage in specific brain regions, which could be developed into a clinical battery for early detection of SAH damage in humans, potentially improving early treatment and outcomes.

## Introduction

Aneurysmal subarachnoid hemorrhage (SAH) is a devastating disease that frequently results from the sudden rupture of an intracranial aneurysm, with high rates of death or severe disability [1, 2]. Survivors of SAH often experience significant neuropsychological impairment, including sensorimotor deficits, cognitive slowing, memory impairment, and language dysfunction [1, 3–6]. Even those with favorable functional outcomes may develop disabling constitutional impairments such as fatigue, depression, and anxiety [7, 8]. These deficits are attributed to both early and ongoing SAH-induced damage [9, 10]. Despite tremendous research efforts spanning decades, nimodipine remains the sole FDA-approved treatment for SAH patients, with marginal benefits at best [11, 12]. No other agents have been shown to reliably improve outcomes for SAH patients [13]. As the search for effective therapies for SAH continues, early detection of salvageable tissue may help guide targeted interventions aimed at preventing long-term deficits.

Historically, angiographic vasospasm has been regarded as the primary cause of SAH-induced damage after the acute phase of injury [14]. The failure of vasospasm treatment to reliably improve long-term outcomes has led researchers to explore other contributors to death and chronic dysfunction following SAH [15]. Unfortunately, most SAH-induced damage is only clinically detected in the late phase of injury, after irreversible damage has occurred [16, 17]. The presence of a new deficit on clinical neurologic examinations can be highly predictive of ongoing ischemic insults [18], but by this point, the damage may already be permanent [19]. In animal models, abnormal behavioral outcomes after SAH are associated with inflammation in specific brain regions without apparent lesion development [20, 21], indicating that these assessments may detect early signs of ongoing damage that are overlooked or unrecognized by standard examination. Thus, it is possible that a comprehensive battery of assessments and re-assessments may accurately detect regional cerebral damage, even at early time points after SAH. However, the overall performance in behavioral tests, including accuracy, sensitivity for detecting regional damage and specificity for damage in particular anatomic regions, has not been established in SAH.

Our study hypothesized that a comprehensive behavioral battery might be a sensitive method for early detection of SAH-induced damage in specific cerebral regions. To test this hypothesis, SAH was induced in rats, using the endovascular filament perforation model, which is considered to most closely mimic aneurysm rupture in humans [22]. A behavioral battery was then performed at multiple time points, followed by histologic analysis of potentially affected regions. The degree of neurobehavioral impairment was compared to the degree of SAH-induced cellular damage in matched cerebral regions to establish the accuracy, sensitivity, and specificity of behavioral testing. Distinct patterns of neurobehavioral deficits were observed after SAH and were found to accurately predict cellular injury in specific anatomic regions. These findings demonstrate that SAH-induced regional cerebral damage can be predicted using specific behavioral test parameters. Adaptation of clinical correlates of these behavioral tests may enable early and accurate prediction of SAH-induced damage in specific regions in humans. Early detection of SAH-induced damage increases the window of opportunity for meaningful intervention, potentially preventing some of the disabling long-term consequences of SAH.

## Materials & Methods

### Animals:

All procedures were performed in accordance with the National Institutes of Health and ARRIVE guidelines for animal research. Experimental protocols were approved by the Institutional Animal Care and Use Committee of the Feinstein Institute for Medical Research, Manhasset, NY (Protocol #2019-018). A total of 30 adult male Sprague-Dawley rats (Taconic Biosciences Inc, Germantown, NY), weighing 325 to 375 g, were housed in temperature-controlled conditions with 12-h light/dark cycle and ad libitum access to food and water. Cages were lined with Enrich-o’Cobs bedding (The Andersons, OH) and enriched with semi-translucent acrylic tubes.

### SAH model:

The endovascular perforation model of SAH was performed as previously described [23]. Briefly, general anesthesia was induced with isoflurane after which animals were transferred to a temperature-controlled heating pad. The surgical field was shaved, sterilized and a midline incision made superior to the sternum. The left common carotid artery (CCA) was exposed, and clips were applied to the proximal CCA, distal internal carotid artery (ICA), and left external carotid artery (ECA). The ECA was ligated and cut to form a vascular stump. A sharpened 3 − 0 Prolene suture was inserted into the stump, the ICA clip was removed, and the suture was advanced to the ICA bifurcation. In the SAH group, the bifurcation was punctured to induce SAH. The suture was then removed, the stump ligated, incision closed, and the animal was placed in a clean cage for observation. In the sham group, the same procedure was performed without puncture of the bifurcation.

### Experimental groups and timeline:

**Figure 1:**
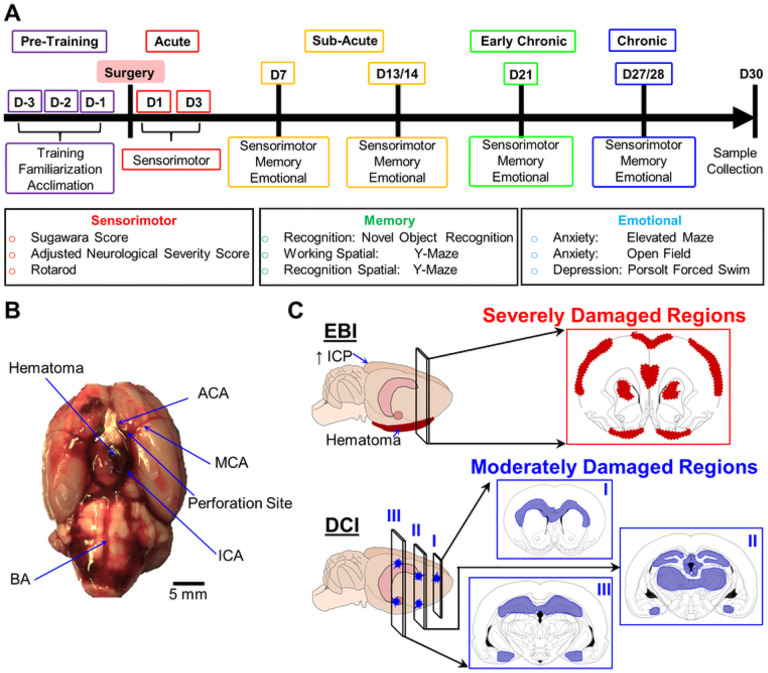
Conceptual diagram of experimental protocols. **A:** Timeline of behavioral testing. After a pre-training period before SAH induction or sham surgery, damage in the brain regions affected by SAH is assessed with a behavioral battery in which the indicated tests measure sensorimotor, memory and affective domains. (**B**) Subarachnoid hemorrhage is induced via endovascular perforation at the internal cerebral artery (ICA) bifurcation using a sharpened suture, after which hematoma forms in the basal subarachnoid space. Other abbreviations: ACA, anterior cerebral artery; BA, basal artery; MCA, medial cerebral artery (**C**) The top panel shows the early brain injury (EBI), which causes severe damage (**red**), and is associated with hematoma formation in the basal subarachnoid space damaging the piriform cortex, as well as increased intracranial pressure (ICP) damaging the motor, somatosensory, and piriform cortices, thalamus, and striatum. The lower panel shows the delayed cerebral ischemia (DCI), with more moderate damage (**blue**) developing in the hippocampus, white matter tracts, amygdala, and periventricular nuclei.

A total of 10 animals underwent sham surgery, and 20 animals underwent SAH induction. In the SAH group, 8 animals died within 72 h of SAH induction and one at 23 days. No sham animals died prior to the experimental endpoint. Behavioral assessments were performed as described below at 24 h (termed 24H henceforth), 72 h (72H), 7 days (7D), 14 days (14D), 21 days (21D), and 28 days (28D). All animals underwent sensorimotor testing at 24H to confirm successful SAH induction or sham surgery. One animal from the SAH group was excluded from analysis due to procedure failure. No rats from the sham group were excluded. At 30 days, animals were euthanized, and samples were taken for histopathologic analysis. Within this experimental model, the period from day zero to 72 h was defined as the acute period, 72 h to 14 days as the subacute period, 14 to 21 days as early chronic, and 21 + days as chronic (Fig. 1A).

### Behavioral assessments:

The tests were performed in a dedicated suite, with consistent lighting and minimal extraneous stimuli, by two technicians blinded to experimental groups, at the same time of day, in order of least stressful to most stressful. Behavioral assessments were done using established protocols, described in detail in the Supplemental Methods.

### Brain tissue preparation:

At 30 days following SAH induction or sham surgery, rats were deeply anesthetized with isoflurane. Once appropriate anesthesia was confirmed, they were transcardially perfused with cold phosphate-buffered saline (PBS), followed by 4% paraformaldehyde (PFA) in PBS. The brains were removed and fixed in PFA overnight, then cryopreserved in gradient sucrose solution, embedded in a 1:3 mixture of 30% sucrose and Optimal Cutting Temperature Compound (Electron Microscopy Sciences, Hatfield, PA) and stored at −80°C. Brains were serially cryo-sectioned (Leica Biosystems, Wetzlar, Germany) as 18-μm thick coronal sections with 400 μM intervals from caudal to rostral. Sections were mounted on Superfrost Plus glass slides (Thermo Fisher Scientific, Waltham, MA) and Polysine glass slides (Thermo Fisher Scientific, Waltham, MA), and stored at −20°C.

### Histological analysis:

For identification of regions of interest (ROI), samples mounted on Superfrost Plus slides were stained with hematoxylin and eosin (H&E) and imaged using PathScan Enabler (Meyer Instruments, Houston, TX) at 2500 dpi with an adjusted brightness of + 30. Images were converted to weighted 8-bit grey scale and processed using ImageJ (ImageJ v153, U. S. National Institutes of Health, Bethesda, Maryland, USA). The Threshold tool was used to assess potential lesion areas after converting images to binary black and white pixels. On H&E staining healthy brain tissue displays a left-skewed pixel intensity distribution reflecting predominantly basophilic staining while eosinophilic staining lesioned tissue has a right-skewed distribution. Anatomic regions were manually segmented using the Waxholm Space atlas [24] to allow for region-to-region comparison. A prespecified minimum pixel intensity threshold (145–254, black) was used, derived from the average intensity of visually identified lesion areas on prior sets of training slides. Values below threshold (1–144, white) were considered the color intensity of healthy brain tissue based on mean values in corresponding anatomic regions of sham animals. Total ROI was manually selected based on area above threshold. The Wand tool was used to select densely packed areas of potential damage, defined as severe potential damage. ROI containing pixel density below threshold for severe potential damage and above 144 was defined as moderate potential damage. ROI of severe and moderate potential damage were quantified as area percentage of the total anatomic area.

After ROI identification, H&E-stained slides were imaged using EVOS M7000 (Thermo Fisher Scientific, Waltham, MA) at low power (20x = 0.2mm^2^) and high power (40x = 0.05mm^2^). All animals from each group underwent quantification of regional cellular health. Five high-power fields were selected at random from within each ROI in SAH group animals and corresponding regions in sham animals. Cellular morphology was compared between groups, and cells categorized as healthy/unhealthy using regional rodent pathology references [25]. To account for loss of severely damaged cells by 30D, the area density of healthy cells was also quantified for each region. Due to the lack of established definitions for SAH-induced lesions, existing literature on ischemic tolerance was used to define lesion severity. A level of 20% unhealthy cells was set as the baseline for moderate damage based on established CBF thresholds for significant prolonged ischemia [26]. As brain tissue can tolerate marked reductions in blood flow for brief periods of time [26, 27], we defined severe damage as a region with greater than 70% unhealthy cells [28]. ROI from SAH and sham animals were assessed and classified as predominantly healthy (less than 20% unhealthy cells), moderately damaged (20–70% unhealthy cells), and severely damaged (> 70% unhealthy cells).

### Statistical analysis:

Statistical tests were performed using the Statistics-and-Machine-Learning Toolbox in MATLAB (version 2022a, MathWorks, Inc. Natick, MA), OriginPro (version 2022b 64-bit, OriginLab, Northampton, MA) and GraphPad Prism (version 9, GraphPad Software, Boston, MA). As all comparisons were between SAH and sham groups, effect sizes and comparisons were performed using a mixed-effects model with an ANOVA. For all tests, group and day were treated as fixed effects while the subject was treated as a random effect. For object exploration time, the effect of treatment as well as the interaction between treatment and object investigated were tested. Post-hoc analysis was performed with the Tukey test. Results are presented as mean ± standard deviation (SD). Statistical significance was set to *P* < 0.05. To measure the sensitivity and specificity of early neurobehavioral deficits to predict long-term regional damage, behavioral test results at earliest assessment were compared to the damage observed at 30D after SAH. The degree of impairment observed in each behavioral assessment parameter was normalized to sham animal performance and compared to the average percentage of healthy cells in associated cerebral regions of the same animal and correlation coefficients calculated. For each region, the best correlated neurobehavioral impairment was selected for receiver operator characteristic (ROC) analysis. Behavioral test accuracy was reported as area under the ROC curve (AUC), and for each assessment a cut-off value with maximum sensitivity and specificity for detecting region-specific cellular damage was identified.

## Results

### Evaluation of SAH-induced regional damage

**Figure 2:**
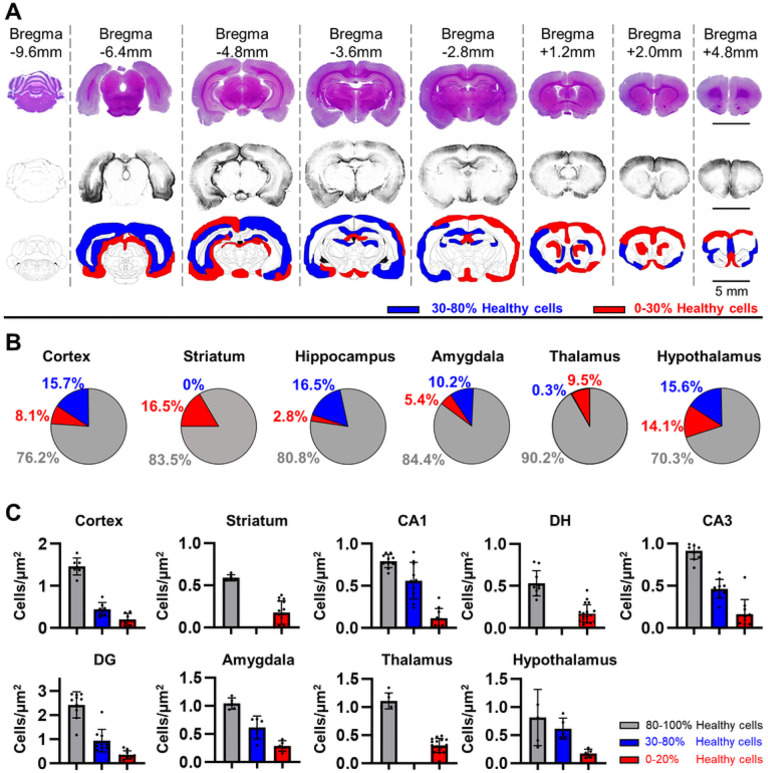
Distribution of damage severity following SAH. **A:** At 30 days following SAH, damage was observed to occur in multiple areas including the cortices, hippocampus, thalamus, hypothalamus, striatum and amygdala, with both severe damage (**red**) defined as more than 70% unhealthy cells and moderate damage (**blue**) defined as 20–70% healthy cells present in most regions assessed. Black bar = 5mm. B: The relative distribution of severe and moderate potential damage in different brain regions was quantified. Most regions displayed 10–30% area of potentially unhealthy regions, with a mixture of severe and moderate potential damage generally favoring moderate damage in all regions except the thalamus and striatum. **C:** The density of healthy cells corresponded to regions of potential damage observed previously. The cortex, hypothalamus, and amygdala exhibited both moderate and severely decreased healthy cell density. Hippocampal regions CA1, CA3 and DG contained both moderate and severe reductions in healthy cell density, while the DH region was entirely severely damaged. The striatum and thalamus also showed severe reductions in healthy cell density.

The experimental design evaluated a rat SAH model to test the sensitivity and specificity of a behavioral battery in detecting cerebral damage at different time points after SAH (Fig. 1A). The endovascular perforation model of SAH causes acute hemorrhage from the ICA bifurcation (Fig. 1B), forming a clot within the basal subarachnoid space [23, 29]. In our model, hemorrhage was found within the basal cisterns at 24H after endovascular perforation (Fig. 1B). At 30 days, lesions were observed throughout the motor cortex, somatosensory cortex, piriform cortex, hippocampus, thalamus, hypothalamus, amygdala, and striatum (Fig. 1C; Fig. 2A). A variable distribution in the moderate-to-severe lesion scale was observed on ROI analysis (Fig. 2B). Several brain regions contained a higher percent of moderate injury, such as the cerebral cortex (moderate: 15.7 ± 5.6%, severe: 8.1 ± 2.9%), hypothalamus (moderate: 15.6 ± 9.6%, severe: 14.1 ± 8.7%), hippocampus (moderate: 16.5 ± 6.0%, severe: 2.8 ± 1.0%), and amygdala (moderate: 10.2 ± 10.7%, severe: 5.4 ± 5.7%). Conversely, the thalamus had almost entirely severe damage (moderate: 0.3 ± 0.1%, severe: 9.5 ± 4.6%), while the striatum contained only severe damage (moderate: 0%, severe: 16.5 ± 9.2%).

### Sensorimotor impairment predicts cortical and striatal damage

**Figure 3:**
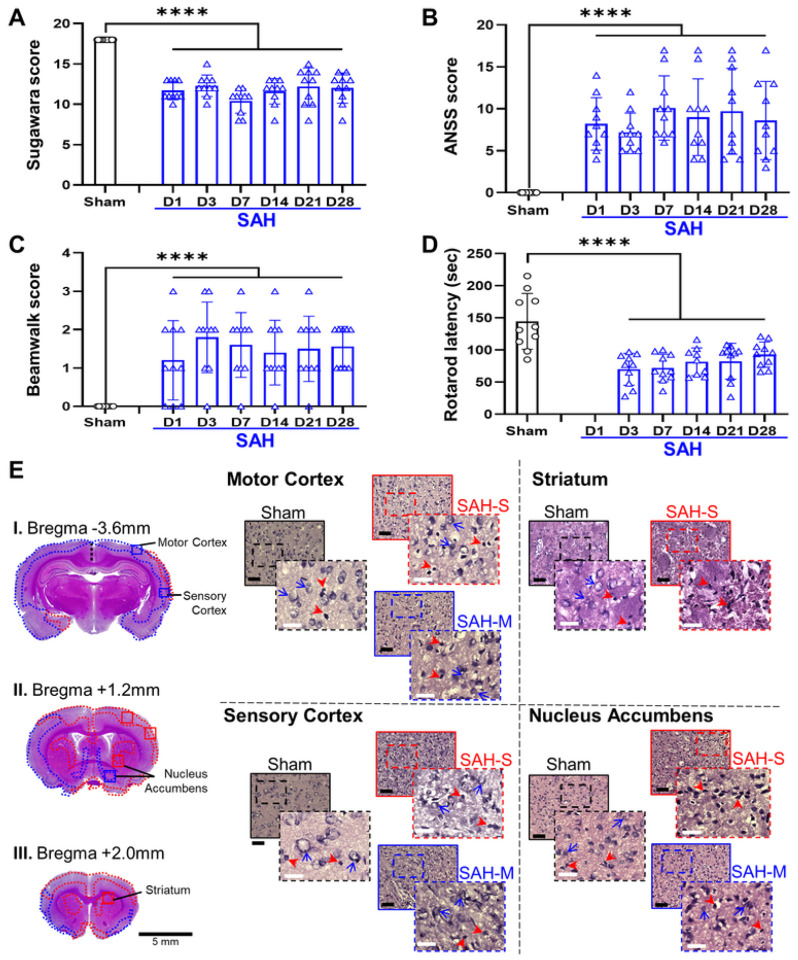
SAH induces sensorimotor dysfunction associated with both moderate and severe damage in the cortex and basal ganglia. **A:** The Sugawara score, a measure of general sensorimotor function following SAH, was significantly decreased, consistent with sensorimotor impairment. **B:** Adjusted neurologic severity score (A-NSS) was elevated, indicating worsened sensorimotor function in SAH animals. **C:** Rotarod latency, or riding time prior to falling, was decreased in the SAH group indicating impaired coordination and sensorimotor ability. **D:** Beam-walk score, a measure of coordination and sensorimotor function, was elevated in rats following SAH, indicating impaired sensorimotor function. SAH rats displayed significantly worsened sensorimotor function at all time points relative to sham baseline. **E:** Severe levels of cellular death (**red outline**) were observed in the motor and somatosensory cortices, along with the striatum. Additionally, regions of moderate damage were also observed in the motor, somatosensory and piriform cortices (**blue outline**) as well as the striatum and nucleus accumbens regions of the basal ganglia. Black bar = 100μm; white bar = 25μm; red arrowheads = unhealthy cells; blue arrows = examples of healthy cells; solid outline = 20x = 0.2mm^2^; dashed outline = 40x = 0.01mm^2^; **** *P* < 0.0001, mixed-effects ANOVA.

The Sugawara score showed no differences between groups at baseline (Fig. 3A; Sham: 18 ± 0, SAH: 18 ± 0). Moreover, the score for the sham group did not change after surgery. The SAH rats showed a significant decrease in Sugawara scores compared to sham (Fig. 3A; 24H: 11.70 ± 1.16, 72H: 12.30 ± 1.34, 7D: 10.40 ± 1.51, 14D: 11.60 ± 1.58, 21D: 12.20 ± 2.30, 28D: 12.00 ± 1.89; *F* = 99.8, *P* = 1.3×10^− 14^, mixed-effects ANOVA). Post-hoc comparisons revealed that the SAH scores were not statistically different across days (*F* = 2.95, *P* = 0.02, Tukey test) except for 72H vs. 7D (*t* = 4.7, *P* = 0.019, Tukey test). Our results demonstrate that significant sensorimotor impairment occurs as early as 24H post-bleed and does not improve by 28D after SAH.

The adjusted-neurological severity score (A-NSS) showed no differences in baseline values (Fig. 3B; Sham: 0 ± 0, SAH: 0 ± 0). The A-NSS remained at zero for the sham group after sham surgery. After induction, SAH animals demonstrated a significant increase in A-NSS as compared to sham (Fig. 3B; 24H: 8.20 ± 3.12, 72H: 7.20 ± 2.30, 7D: 10.10 ± 3.84, 14D: 9.00 ± 4.57, 21D: 9.70 ± 5.12, 28D: 8.60 ± 4.67; *F* = 28.7, *P* = 1.26×10^− 6^, mixed-effects ANOVA). The A-NSS beam-walk portion assessing balance and coordination showed significant increases in the SAH animals (Fig. 3C; 24H: 1.20 ± 1.03, 72H: 1.80 ± 0.91, 7D: 1.60 ± 0.84, 14D: 1.40 ± 0.84, 21D: 1.50 ± 0.85, 28D: 1.50 ± 0.53; *F* = 1.38, *P* = 1.92×10^− 5^, mixed-effects ANOVA). There were no significant differences in A-NSS scores in the SAH group between time points (*F* = 0.45, *P* = 0.81). These results suggest that SAH induces sensorimotor dysfunction, without improvement in the acute period and beyond.

For the rotarod test, there was a significant decrease in riding time in SAH animals compared to sham (Fig. 3D; 72H: 70.00 ± 24.86 s, 7D: 72.20 ± 21.37 s, 14D: 81.67 ± 21.51 s, 21D: 82.50 ± 27.95 s, 28D: 92.90 ± 16.66 s; *F* = 27.68, *P* = 2.52×10^− 6^, mixed-effects ANOVA). No significant variation in riding time was observed across days in the SAH group (*F* = 1.69, *P* = 0.17). These results suggest that SAH impairs motor function and coordination in a long-lasting manner.

Cellular health in the motor and sensory cortices was in the severe range (Fig. 3E; Sham: 82.8 ± 6.56%, SAH: 15.7 ± 14.7%, *P* = 2.19 ×10^− 11^) and the density of healthy cells in the cortex was significantly reduced (Fig. 2C; Sham: 1.55 ± 0.219 cells/μm^2^; SAH: 0.398 ± 0.255 cells/μm^2^; *P* = 1.94×10^− 12^). The striatum displayed a significant degree of severe damage (Fig. 3E; Sham: 71.4 ± 2.03%, SAH: 24.9 ± 22.9%, *P* = 3.2×10^− 6^), as well as a significant reduction in healthy cell density (Fig. 2C; Sham: 0.569 ± 0.0487 cells/μm^2^; SAH: 0.221 ± 0.154 cells/μm^2^; *P* = 1.59 ×10^− 7^).

Sensorimotor assessments corresponded well with damage observed in regions implicated in sensorimotor function. The A-NSS was highly correlated with cortical damage (R^2^ = 0.907) but less with striatal damage (R^2^ = 0.736). The Garcia scores correlated equally with damage to the cortices (R^2^ = 0.849) and striatum (R^2^ = 0.826). Rotarod maximum speed correlated better with damage to the cortices (R^2^ = 0.875) than striatum (R^2^ = 0.808), while rotarod riding time correlated better with striatal health (R^2^ = 0.819) than cortical health (R^2^ = 0.679). The A-NSS was selected for ROC analysis (Fig. 9A) demonstrating that a cut-off score of 2 for the A-NSS provided optimal sensitivity and specificity for predating severe cortical damage (AUC: 0.905; 95% CI: 0.742–1.00; sensitivity: 81.8%; specificity: 90.9%). The Garcia score was best correlated with striatal health by ROC analysis (Fig. 9B) demonstrating a cut-off score of 13 for optimal sensitivity and specificity (AUC: 0.913; 95% CI: 0.750–1.00; sensitivity: 90.9%; specificity: 100%).

### Memory impairment predicts hippocampal damage

**Figure 4:**
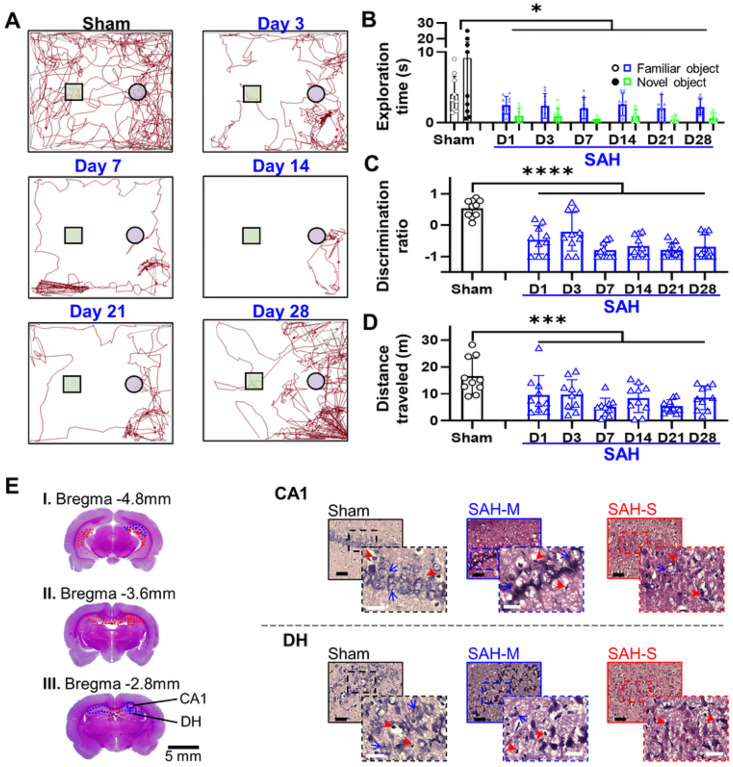
SAH induces working reference memory dysfunction on Novel Object Recognition (NOR) assessment associated with predominantly moderate cellular damage in hippocampal regions CA1 and the dentate hilus (DH). **A:** Representative traces demonstrating reduced movement and interaction in SAH animals (square = novel object, circle = familiar object). **B:** The amount of time SAH rats spent exploring either object was diminished, suggesting impaired sensorimotor and exploratory function. SAH rats also spent less time investigating the novel object relative to time spent investigating the familiar object. **C:** Discrimination ratio was decreased in animals following SAH, consistent with impaired reference memory. **D:** Total distance moved during the NOR task was reduced in SAH rats. **E:** Examination of the DH and CA1 regions of the hippocampus revealed presence of severe damage (**red outline**) and moderate damage (**blue outline**) with a greater relative proportion of moderate damage. Black bar = 100μm; white bar = 25μm; red arrowheads = unhealthy cells; blue arrows = examples of healthy cells; solid outline = 20x = 0.2mm^2^; dashed outline = 40x = 0.01mm^2^; * *P* < 0.05, **** *P* < 0.0001, mixed-effects ANOVA.

The novel object recognition test (Fig. 4A) showed that the SAH group had a significant decrease in time interacting with either object (Fig. 4B; 24H: 3.49 ± 2.39 s, 72H: 6.62 ± 5.18 s, 7D: 4.37 ± 5.80 s, 14D: 6.27 ± 5.02 s, 21D: 5.59 ± 6.35 s, 28D: 4.30 ± 2.90 s; *F* = 6.2015, *P* = 0.014, mixed-effects ANOVA for treatment and object). Importantly, SAH animals showed a significant reduction in the interaction with the novel object relative to the familiar object (*F* = 19.78, *P* = 1.84×10^− 5^, mixed-effects ANOVA for treatment and object), with a significant decrease in discrimination ratio (Fig. 4C; 24H: −0.465 ± 0.500, 72H: −0.212 ± 0.615, 7D: −0.801 ± 0.244, 14D: −0.670 ± 0.335, 21D: −0.784 ± 0.224, 28D: −0.693 ± 0.379; *F* = 38.55, *P* = 4.68×10^− 8^, mixed-effects ANOVA vs. sham). There was also a significant reduction in the total distance traveled (Fig. 4D; 24H: 9.61 ± 7.24 m, 72H: 9.7 ± 5.57 m, 7D: 5.11 ± 3.31 m, 14D: 8.4 ± 5.35 m, 21D: 5.46 ± 2.44 m, 28D: 8.59 ± 4.44 m, *F* = 9.33, *P* = 0.0033). These findings suggest that SAH induces persistent impairment in recognition memory.

**Figure 5:**
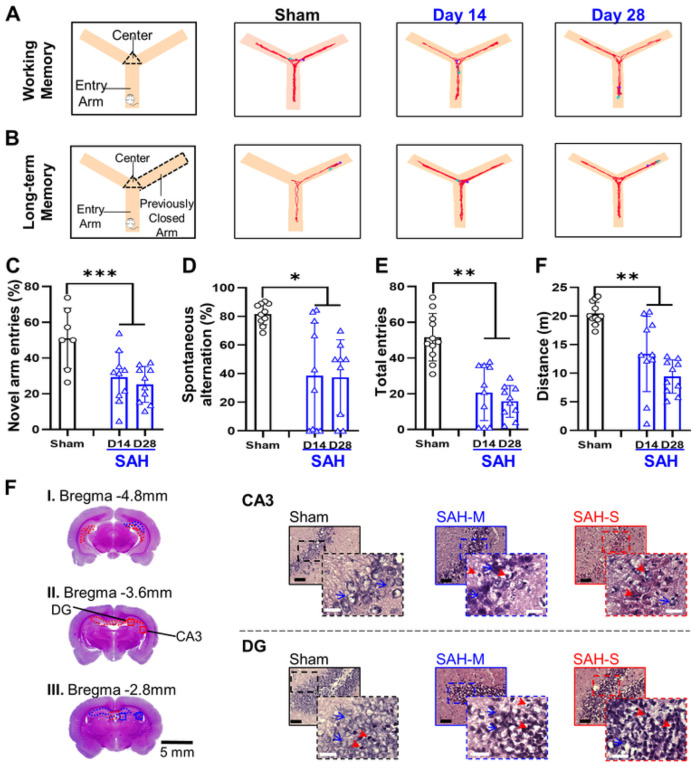
SAH induces working and reference spatial memory dysfunction and decreases exploratory and locomotor behavior on Y-Maze assessment in association with damage in the hippocampal CA3 and dentate gyrus (DG) regions. **A:** Representative traces illustrating impaired working spatial memory **B:** Representative traces demonstrating impaired reference spatial memory. **C:** Decreased entries of the previously closed arm were observed following SAH, consistent with impaired reference spatial memory. **D:** Percentage of complete spontaneous alternations was decreased in SAH animals, indicating impaired working spatial memory. **E:** Rats in the SAH group had fewer total entries into arms, consistent with impaired locomotor and exploratory function. **F:** Total distance travelled during the Y-Maze assessment decreased, indicating exploratory and locomotor function impairment following SAH. **F:** A small degree of severe damage (**red outline**) and larger proportion of moderate damage (**blue outline**) were observed in the CA3 and DG regions of the hippocampus following SAH, which are closely related to formation and retrieval of spatial memory. Black bar = 100μm; white bar = 25μm; red arrowheads = unhealthy cells; blue arrows = examples of healthy cells; solid outline = 20x = 0.2mm^2^; dashed outline = 40x = 0.01mm^2^; * *P* < 0.05, ** *P* < 0.01, *** *P* < 0.001, mixed-effects ANOVA.

The working memory test in the Y maze (Fig. 5A) showed that the SAH group had significantly lower novel arm entries (Fig. 5C; Sham: 51.9 ± 14.2%; SAH: 14D: 31.3 ± 13.6%, 28D: 26.2 ± 10.8%; *F* = 14.1, *P* = 8.44×10^− 4^, mixed-effects ANOVA) as well as lower percentage of successful complete alternations (Fig. 5D; Sham: 71.6 ± 13.1%; SAH: 14D: 39.8 ± 38.3%, 28D: 33.28 ± 27.03%; *F* = 7.1, *P* = 0.012, mixed-effects ANOVA). Moreover, the reference memory test in the Y maze (Fig. 5B) showed that SAH animals had significantly lower total entries to the previously closed arm (Fig. 5E; Sham: 42.3 ± 15.7; SAH: 14D: 21.0 ± 15.72, 28D: 17.3 ± 27.03; *F* = 12.9, *P* = 0.0013, mixed-effects ANOVA) and a significant decrease in the total distance traveled over the course of the test (Fig. 5F; Sham: 19.99 ± 2.68 m; SAH: 14D: 13.36 ± 6.56 m, 28D: 9.43 ± 2.92 m; *F* = 12.46, *P* = 0.0015, mixed-effects ANOVA). These results suggest that SAH induces impairment in working/reference memory in the subacute and chronic phases.

The CA1 area of the hippocampus showed significant cellular damage in the moderate range (Fig. 4E; Sham: 84.2 ± 5.1%, SAH: 30.3 ± 34.3%, *P* = 9.59×10^− 5^), while the dentate hilus (DH;) displayed severe cellular damage (Fig. 4E; Sham: 73.2 ± 4.4%, SAH: 17.9 ± 11.8%, *P* = 9.45×10^− 12^). Moreover, there was significant moderate cellular damage in the CA3 area (Fig. 5F; Sham: 82.4 ± 2.8%, SAH: 34.2 ± 25.1%, *P* = 6.86×10^− 6^) and the dentate gyrus (DG, Fig. 5F; Sham: 86.3 ± 8.2%, SAH: 36.9 ± 24.5%, *P* = 6.13 ×10^− 6^). Quantification of healthy cell density in the hippocampus revealed reduced values in the SAH group compared to sham in all regions (Fig. 2C), such as CA1 (Sham: 0.80 ± 0.08 cells/μm^2^; SAH: 0.34 ± 0.29 cells/μm^2^; *P* = 3.46×10^− 5^), CA3 (Sham: 0.91 ± 0.10 cells/μm^2^; SAH: 0.31 ± 0.21 cells/μm^2^; *P* = 2.99 ×10^− 9^), and DG (Sham: 2.43 ± 0.54 cells/μm^2^; SAH: 0.65 ± 0.46 cells/μm^2^; *P* = 3.17×10^− 10^). Lastly, the DH also showed reduced density of healthy cells (Sham: 0.53 ± 0.15 cells/μm^2^; SAH: 0.18 ± 0.11 cells/μm^2^; *P* = 2.9 ×10^− 8^).

Memory assessments corresponded well with damage to the different areas of the hippocampus. For recognition memory, the NOR discrimination ratio correlated with CA1 health (R^2^ = 0.737) but less so with DH (R^2^ = 0.411). Comparing the discrimination ratio obtained via NOR to CA1 health (Fig. 9C), the ROC analysis demonstrated that a discrimination ratio cut-off value of 0.5 had optimal sensitivity and specificity (AUC: 0.902; 95% CI: 0.699–1.00; sensitivity: 74.1%; specificity: 83.3%). For working memory, the percentage of novel arm entries was found to correlate better with CA3 health (R^2^ = 0.708) than with DG health (R^2^ = 0.392). Spontaneous alternations were observed to correlate well with CA3 health (R^2^ = 0.807) but not with DG health (R^2^ = 0.194). As the best correlated measure of working memory, spontaneous alternations and CA3 health were compared via ROC analysis. A cut-off value of 60% for spontaneous alternations was found to maximize sensitivity and specificity (Fig. 9D; AUC: 0.746; 95% CI: 0.576–0.928; sensitivity: 72.2%; specificity: 58.0%), although test performance was worse than recognition memory.

### Affective impairment predicts subcortical damage

**Figure 6:**
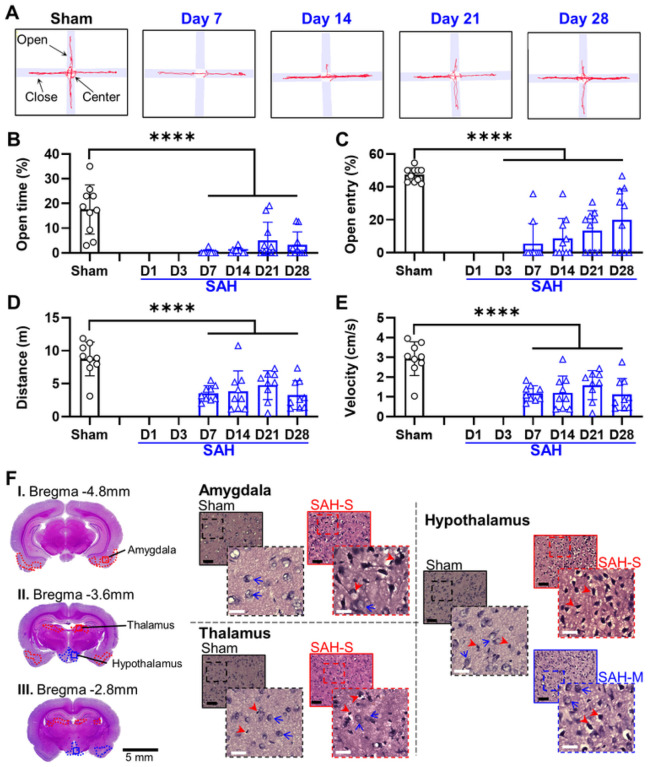
SAH induces anxiety and decreases exploratory and locomotor behavior in the elevated maze (EM) assessment in association with damage affecting the amygdala, thalamus and hypothalamus. **A:** Representative traces illustrate decreased movement into the open arms by SAH rats, with a trend towards improvement over time. **B:** SAH rats spent less time in the open arms indicating increased anxiety-related behavior. **C:** SAH rats made fewer entries into the open arms, consistent with increased anxiety. **D:** Decreased locomotor function was observed in SAH rats, as shown by decreased total distance travelled. **E:** Velocity of movement was also decreased following SAH, indicating impaired locomotor function. **F:** Moderate damage (**blue outline**) was observed in the hypothalamus and amygdala following SAH induction, with severe damage (**red outline**) predominantly observed in the thalamus. Black bar = 100μm; white bar = 25μm; red arrowheads = unhealthy cells; blue arrows = examples of healthy cells; solid outline = 20x = 0.2mm^2^; dashed outline = 40x = 0.01mm^2^; **** *P* < 0.0001, mixed-effects ANOVA.

For the EM test (Fig. 6A), the SAH group demonstrated a significant decrease in the proportion of time spent in the open arms when compared to sham (Fig. 6B; Sham: 17.56 ± 0.87%; SAH: 7D: 0.29 ± 0.87%, 14D: 0.77 ± 1.07%, 21D: 5.06 ± 7.38%, 28D: 3.29 ± 5.18%; *F* = 42.37, *P* = 6.02×10^− 8^, mixed-effects ANOVA), as well as a significant decrease in the percent of open arm entries compared to sham (Fig. 6C; Sham: 47.32 ± 4.21%; SAH: 7D: 5.48 ± 12.20%, 14D: 8.66 ± 12.05%, 21D: 13.38 ± 12.07%, 28D: 19.95 ± 18.83%; *F* = 60.05, *P* = 8.01×10^− 10^, mixed-effects ANOVA). There were no significant variations across days in the time spent in open arms (*F* = 2.29, *P* = 0.103) and the open arm entries (*F* = 2.16, *P* = 0.11) in the SAH group. SAH animals also demonstrated a significant decrease in total distance traveled (Fig. 6D; Sham: 8.77 ± 2.42 m; SAH: 7D: 3.53 ± 1.16 m, 14D: 3.84 ± 3.09 m, 21D: 4.78 ± 2.19 m, 28D: 3.28 ± 2.18 m; *F* = 27.27, *P* = 5.48×10^− 6^, mixed-effects ANOVA) and significantly reduced average speed compared to sham (Fig. 6E; Sham: 2.97 ± 0.81 cm/s; SAH: 7D: 1.27 ± 0.47 cm/s, 14D: 1.34 ± 0.9 cm/s, 21D: 1.5 ± 0.75 cm/s, 28D: 1.14 ± 0.76 cm/s; *F* = 27.55, *P* = 3.99×10^− 6^, mixed-effects ANOVA). These results demonstrate that SAH induces persistent anxiety within 7 days in rats, as well as impairment in locomotor function.

**Figure 7:**
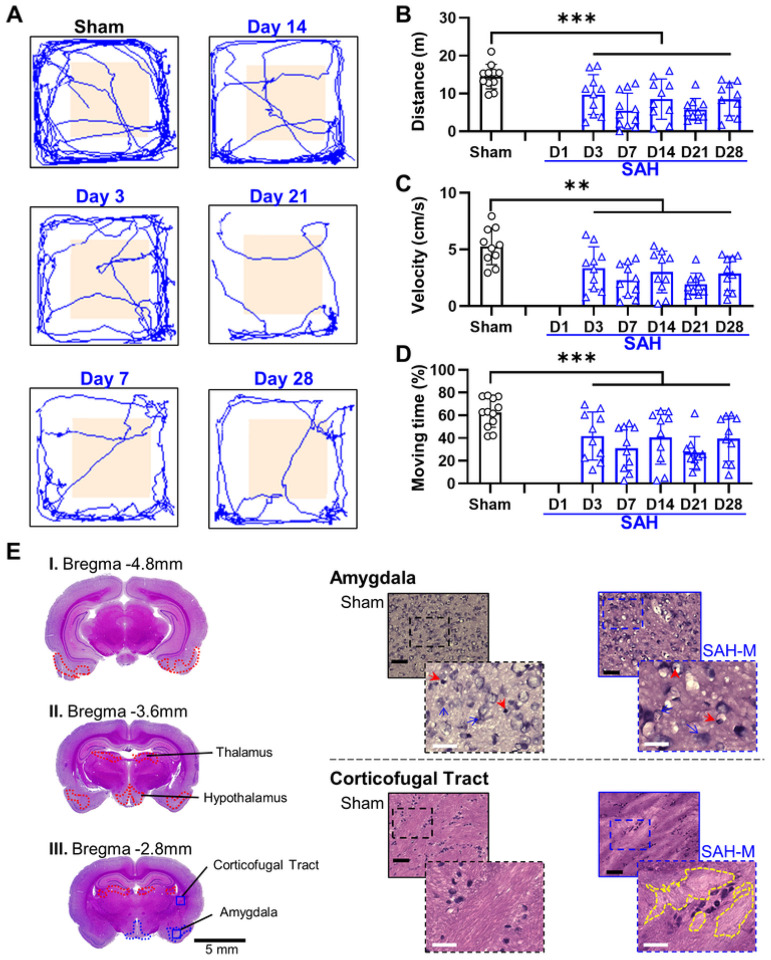
SAH decreases exploratory and locomotor behavior in the open-field (OF) assessment in association with moderate damage in the amygdala and corticofugal tract. **A:** Representative traces illustrating lack of significant change in time in center of field. **B:** There was decreased locomotor activity by rats in the SAH group, as observed by decreased total distance travelled. **C:** Average velocity decreased in SAH group, consistent with locomotor impairment. **D:** Percentage of time spent moving was significantly lower in SAH animals. **E:** Regions of moderate cellular death (**blue outline**) were observed following SAH in the amygdala, along with loss of confluence (**yellow outline**) in the corticofugal tract. Black bar = 100μm; white bar = 25μm; red arrowheads = unhealthy cells; blue arrows = examples of healthy cells; solid outline = 20x = 0.2mm^2^; dashed outline = 40x = 0.01mm^2^; * *P* < 0.05, ** *P* < 0.01, mixed-effects ANOVA.

For the OF test (Fig. 7A), the SAH group showed a reduction in distance traveled compared to sham (Fig. 7B; Sham: 14.4 ± 3.72 m; SAH: 3D: 9.7 ± 5.27 m, 7D: 6.02 ± 4.43 m, 14D: 8.51 ± 5.32 m, 21D: 5.79 ± 2.88 m, 28D: 8.46 ± 4.36 m; *F* = 6.48, *P* = 0.014, mixed-effects ANOVA) and lower mean speed than sham (Fig. 7C; Sham: 5.28 ± 1.61 cm/s; SAH: 3D: 3.36 ± 1.89 cm/s, 7D: 2.29 ± 1.45 cm/s, 14D: 3.02 ± 1.83 cm/s, 21D: 1.94 ± 0.97 cm/s, 28D: 2.88 ± 1.49 cm/s; *F* = 8.22, *P* = 0.0058, mixed-effects ANOVA). Moreover, the SAH group had significantly less time in motion compared to sham (Fig. 7D; Sham: 63.71 ± 13.72%; SAH: 3D: 41.79 ± 21.96%, 7D: 30.92 ± 19.13%, 14D: 40.58 ± 23.7%, 21D: 27.02 ± 14.15%, 28D: 39.4 ± 19.9%; *F* = 7.74, *P* = 0.074, mixed-effects ANOVA). However, there were no significant differences in the proportion of time spent in the center area between groups (Sham: 4.8 ± 3.18%; SAH: 3D: 3.37 ± 2.86%, 7D: 2.49 ± 1.82%, 14D: 4.12 ± 5.19%, 21D: 4.06 ± 4.25%, 28D: 4.21 ± 3.06%; *F* = 0.91, *P* = 0.35, mixed-effects ANOVA). Our results again suggest that SAH induces locomotor dysfunction, however the OF test failed to demonstrate an increase in anxiety-like behavior. Acclimation with the structurally similar NOR arena may have reduced anxiety typically associated with an open, unfamiliar area.

**Figure 8:**
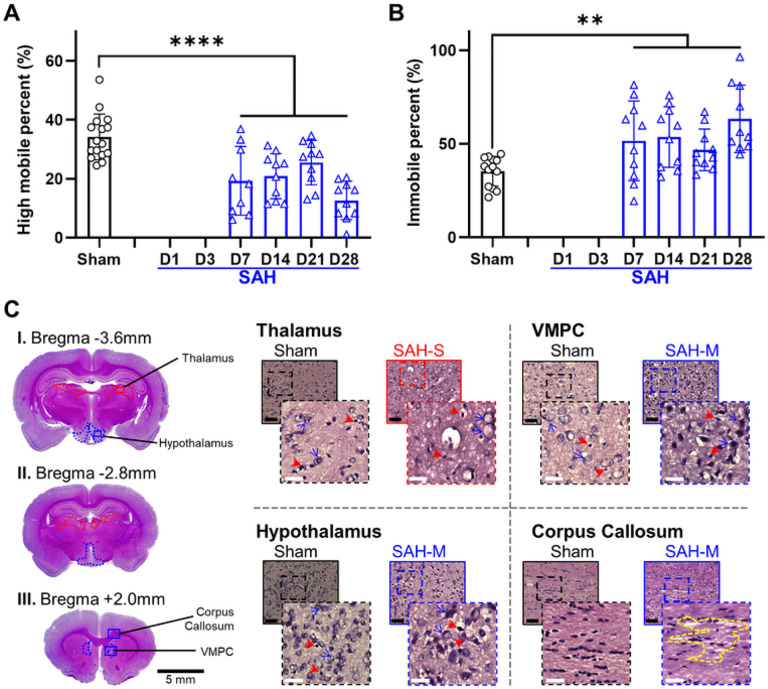
SAH induces situational depression measured via Porsolt Forced Swim (PFS) in association with cortical, subcortical and white matter injury. **A:** SAH group animals showed a significant decrease in highly mobile behavior, indicating a more depressed state. **B:** Following SAH, animals displayed a significant increase in immobile behavior consistent with depression. **C:** Severe damage (**red outline**) was observed following SAH in the thalamus, along with moderate damage (**blue outline**) in the hypothalamus and ventromedial prefrontal cortex (VMPC). Loss of confluence was observed in the corpus callosum (**yellow outline**). Black bar = 100μm; white bar = 25μm; red arrowheads = unhealthy cells; blue arrows = examples of healthy cells; solid outline = 20x = 0.2mm^2^; dashed outline = 40x = 0.01mm^2^; ** *P* < 0.01, **** *P* < 0.0001, mixed-effects ANOVA.

For the FS test, assessing situational depression-like behavior, SAH rats demonstrated a significant decrease in highly mobile time (Fig. 8A; Sham: 37.65 ± 7.78%; SAH: 7D: 19.3 ± 11.65%, 14D: 20.9 ± 7.69%, 21D: 25.5 ± 7.54%, 28D: 12.7 ± 6.48%; *F* = 25.1, *P* = 9.29×10^− 6^, mixed-effects ANOVA), and a significant increase in immobile time compared to sham (Fig. 8B; Sham: 34.01 ± 8.43%; SAH: 7D: 51.6 ± 21.3%, 14D: 53.7 ± 16.2%, 21D: 46.8 ± 11.1%, 28D: 63.3 ± 18%; *F* = 7.28, *P* = 0.0097, mixed-effects ANOVA). The SAH group also demonstrated a significant decrease in highly mobile time (*t* = 5.077, *P* = 0.0075, Tukey test) and increase in immobile time between 21D and 28D time points (*t* = 3.98, *P* = 0.042, Tukey test). Our results suggest that depression begins as early as 7 days following SAH and worsens over the chronic timeframe.

Quantification of cellular health in the amygdala (Fig. 6F, Fig. 7E) revealed moderate range cellular damage (Sham: 82.2 ± 10.2%, SAH: 35.4 ± 15.0%, *P* = 5.71×10^− 8^). Similarly, the density of healthy cells in the amygdala (Fig. 2C) was observed to be reduced compared to sham (Sham: 1.07 ± 0.09 cells/μm^2^; SAH: 0.44 ± 0.23 cells/μm^2^; *P* = 5.94×10^− 9^). The thalamus (Fig. 6F, Fig. 8C) had significant severe cellular damage (Sham: 80.6 ± 9.2%, SAH: 21.8 ± 12.6%, *P* = 9.47×10^− 11^). Healthy cell density in the thalamus (Fig. 2C) was significantly reduced (Shan: 1.14 ± 0.16 cells/μm^2^; SAH: 0.35 ± 0.14 cells/μm^2^; *P* = 1.96×10^− 14^). Lastly, the hypothalamus (Fig. 6F, Fig. 8C) displayed a significant degree of severe cellular damage (Sham: 54.3 ± 26.3%, SAH: 18.7 ± 12.5%, *P* = 0.000477) and a significant reduction in healthy cell density compared to sham (Fig. 2C) (Sham: 0.82 ± 0.46 cells/μm^2^; SAH: 0.45 ± 0.24 cells/μm^2^; *P* = 0.00582). Additionally, a loss of normal confluence was appreciated in the corpus callosum (Fig. 8C) and corticofugal tract (Fig. 7E), indicating damage to white matter structures.

**Figure 9:**
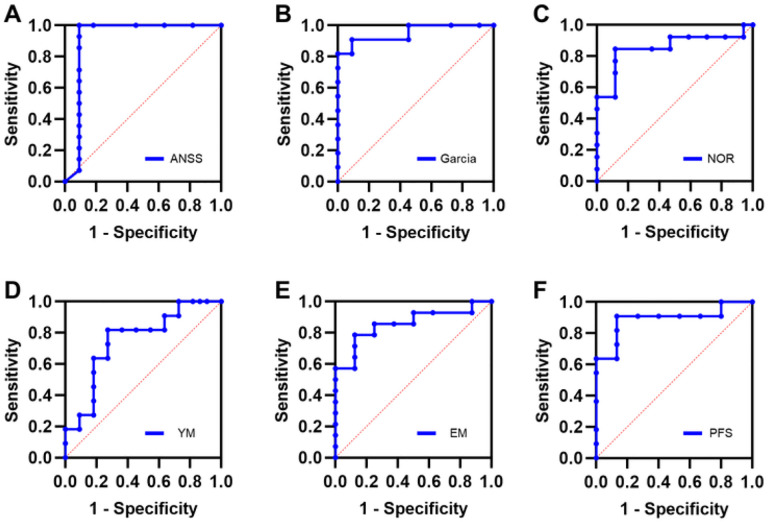
Neurobehavioral tests predict regional cellular damage after SAH. For each region, neurobehavioral tests were chosen that best correlated with the degree of cellular damage observed in each region. **A:** The A-NSS score was observed to correlate best with regional damage in the cortices, and accurately predicted cortical damage with high sensitivity and specificity. **B:** The Garcia score correlated better with striatal damage and demonstrated high sensitivity and specificity to accurately detect damage to the striatum. **C:**The NOR test was observed to be best correlated with damage in the CA1 region of the hippocampus and was able to accurately detect CA1 regional damage. **D:** The YM test correlated best with damage in the CA3 region of the hippocampus but was less accurate than the NOR in detecting hippocampal damage. **E:** The EM test correlated best with cellular health in the amygdala and was able to accurately detect and localize damage to the amygdala. **F:**The PFS was best correlated with thalamus cellular health and demonstrated the ability to accurately detect damage in this region.

Open arm time on EM testing correlated with health in the amygdala (R^2^ = 0.705) but not the thalamus (R^2^ = 0.022) or hypothalamus (R^2^ = 0.057). Similarly, EM open arm entries were better correlated with amygdala health (R^2^ = 0.560) than thalamus (R^2^ = 0.031) or hypothalamus (R^2^ = 0.042). The percentage of open arm time on EM was compared to the health of the amygdala, with a cut-off value of 20% time in open arms maximized prediction of amygdala damage (Fig. 9E) (AUC: 0.900; 95% CI: 0.713–1.00; Sensitivity: 77.0%; Specificity: 81.7%). No correlation was observed between the percentage of center time on OF testing and amygdala health (R^2^ = 0.079). Despite this, a cut off value of 4% center time on OF testing could be identified that provided reasonable accuracy (AUC: 0.888; 95% CI: 0.741–0.956; Sensitivity: 75.9%; Specificity: 83.6%). Highly mobile behavior on PFS was found to be well correlated with cellular health in the thalamus (R^2^ = 0.820) but not the hypothalamus (R^2^ = 0.006). Immobile behavior on PFS was also well correlated with decreased cellular health in the thalamus (R^2^ = 0.851) but not the hypothalamus (R^2^ = 0.006). ROC analysis (Fig. 9F) found a cut-off value of 50% immobility was optimal for predicting severe damage in the thalamus (AUC: 0.963; 95% CI: 0.744–1.00; Sensitivity: 86.3%; Specificity: 87.8%).

## Discussion

Detecting patients at risk for delayed neurologic deterioration is a major challenge in the clinical care of SAH [30]. This period of ongoing injury is regarded as the most significant reversible factor in a patient’s recovery [31]. While vessel imaging techniques can identify large vessel vasospasm, other critical drivers of SAH-induced damage and dysfunction are difficult to detect [18, 32, 33]. Early identification and prediction of ongoing cerebral damage may be crucial for the development of effective therapies for SAH, but no reliable method currently exists to do so [18]. Neurobehavioral deficits have been linked to inflammation, white matter disruption, and other factors that contribute to SAH-induced damage, suggesting that they could serve as a harbinger of permanent cerebral injury [20, 21]. Furthermore, comprehensive behavioral assessments have the potential to detect subtle signs of cerebral damage that are independent of more obvious neurological deficits and can be detected earlier than some imaging methods may reliably identify regional damage [34]. These behavioral assessments provide an opportunity for early and sensitive detection of dysfunctional cerebral regions consistent with SAH-induced damage. Our experimental results have shown that early neurobehavioral deficits accurately predict cellular injury within brain regions. Specifically, sensorimotor impairment, recognition memory impairment, anxiety-like and depression-like behaviors predicted cellular injury in the cerebral cortex and striatum, hippocampus, amygdala, and thalamus, respectively, with good accuracy (> 90%). Spatial memory impairment predicted hippocampal damage with acceptable accuracy (~ 75%). No deficit was found to predict damage to the hypothalamus.

Sensorimotor deficits were found to be sensitive and specific indicators of SAH-induced regional damage, involving complex processes in the cortex, basal ganglia, thalamus, white matter tracts, and brainstem loci [35]. In this study, severe damage was observed in the cortex, basal ganglia and thalamus, with moderate potential damage ranging from 15.7% in the cortices to 0.3% in the thalamus. Similarly, cellular health was observed to be severely damaged in all animals but ranged from an average of 15.7% in the cortex to 24.9% in the striatum. Most parameters of sensorimotor assessments correlated well with cellular damage in cortical and subcortical regions known to be involved in sensorimotor function. Specifically, the 24H A-NSS and Garcia scores were the best indicators of damage to the cortices and striatum, respectively, with accuracies greater than 90%. These findings suggest that sensorimotor impairment is highly sensitive and specific for damage in the cortex and striatum. While associations between sensorimotor impairments and cellular health in the acute phase of SAH have been reported by others [36], our study provides novel findings of similar correlations in the chronic phase. While the study did not investigate the mechanisms underlying cellular damage, it is possible that the observed sensorimotor deficits are caused by a combination of early ischemia and ongoing inflammation, microvascular spasm and/or microthrombosis [37–39]. These factors may contribute to sensorimotor impairment beyond ischemic infarctions and highlight the complex nature of SAH-induced damage.

In this study, memory deficits were correlated with damage in associated hippocampal regions, with the NOR test appearing more accurate than the Y-maze test. There is wide agreement that CA1, CA3 and the dentate gyrus play essential roles in encoding memory [40–44]. When assessed histologically, most regions of the hippocampus displayed a greater degree of moderate potential damage than severe damage, with only the DH having a percentage of healthy cells below 30%. Significant early memory impairment was observed, which worsened over time and correlated with damage in the CA1 and CA3 regions. The NOR test accurately predicted cellular damage in CA1, while the Y-maze was somewhat less accurate in predicting damage to CA3. Previous studies have demonstrated that selective knockout of hippocampal neurons causes specific patterns of memory impairment [41, 45]. However, others have demonstrated a variable relationship between neurobehavioral deficits and cellular loss in SAH models [46, 47], with some proposing that memory deficits are driven by alterations in long-term potentiation rather than direct cellular damage [20, 48]. Our results suggest that the NOR test is most sensitive and specific for detecting hippocampal damage associated with SAH, which alongside likely alterations in neuroplasticity led to long-term memory impairment.

We found that measures of anxiety-like behavior were highly sensitive and specific predictors of damage to the amygdala. Although anxiety is a complex psychological condition, the amygdala is known to play a role in pathologic anxiety in various disease states [49, 50]. Here we predominantly observed moderate potential damage as well as moderate cellular damage in the amygdala. Early anxiety-like behavior was present in the EM test although it showed a trend towards improvement over time. Conversely, the OF test did not demonstrate significant anxiety-like behavior at any time point. The percentage of open arm entry for the EM demonstrated a moderate (> 70%) correlation with and accuracy for predicting damage to the amygdala. While anxiety-like behavior has been frequently reported in experimental models of SAH, no preclinical studies have directly linked SAH-related anxiety to cellular damage in the amygdala [51–53]. However, anxiety is a common feature in patients who survive SAH [54], and clinical studies have demonstrated dysfunction in the amygdala in SAH patients [55]. This reinforces that behavioral assessments of anxiety-like behavior may be sensitive and specific for detecting dysfunction and damage affecting the amygdala and may provide a useful tool for predicting the development of damage in the amygdala.

Depression is a common and disabling condition that often occurs after SAH [56], and our study found that measures of depression-like behavior are both sensitive and specific for predicting damage in associated cerebral regions. Although the anatomic correlates of post-SAH depression are not well understood, studies in patients with SAH have demonstrated dysfunction in the cingulate cortex, which has also been observed in preclinical and clinical studies of depression [55, 57, 58]. Additionally, the thalamus and hypothalamus have been implicated in depression in humans [59, 60], although their contribution to depressive behavior in SAH is less clear. Here the hypothalamus demonstrated a large degree of moderate and severe potential damage and the thalamus contained almost entirely severe potential damage. Cellular health was observed to be severe in both regions. SAH animals displayed severe and worsening depression-like behavior in the PFS, consistent with the damage observed in these structures. The degree of immobile behavior correlated with thalamic health, accurately predicting long-term damage to the thalamus. Other authors have found that animals that lacked significant depression-like behavior following SAH also had no measurable damage in the thalamus [61], supporting the utility of behavioral tests of depression-like behavior in identifying damaged cerebral regions.

We show that a comprehensive battery of behavioral assessments is a sensitive means to identify and predict damage and dysfunction in specific regions after SAH. The sensitivity and specificity of any individual behavioral assessment might vary, but applying multiple combined assessments interrogating the same region can increase sensitivity or specificity [62]. For example, combining the A-NSS and Garcia assessments in parallel allows for a combined sensitivity of 98.8% for detecting damage in structures critical for sensorimotor function, at the expense of a reduced specificity of 89.6%. This technique may be particularly useful with tests of anxiety-like and depression-like behaviors, which can be more affected by variability in animal behavior than other behavioral assessments [52, 63]. While the accuracy of the EM assessment of anxiety was good, the sensitivity for detecting damage in the amygdala could be augmented via combination with another assessment such as the light/dark box test [64], although this test has not been applied in SAH models. In clinical practice, delayed, new-onset sensorimotor deficit usually portends impending irreversible injury, and its rapid recognition allows for treatment that greatly improves neurological outcomes after SAH [65, 66]. While the comprehensive behavioral battery used in this study would likely be effective in detecting and localizing SAH-induced damage, it may not be feasible for regular clinical use. Therefore, it is crucial to identify the most sensitive and specific behavioral tests for damage to specific brain regions. By linking each at-risk region to a specific behavioral test parameter that accurately predicts long term damage, clinical exam correlates of these test parameters could be identified and refined to reliably detect reversible damage before it becomes permanent. These techniques may enable the rapid and accurate identification of patients at risk for irreversible injury, thereby improving outcomes after SAH.

Several limitations exist within this study. H&E staining is an established means of quantifying general cellular damage following neurologic insults [67–69], however the use of cell-specific staining techniques may provide more detailed information regarding the mechanisms underlying ongoing cellular damage. Histological analysis was chosen at the 30-day point to better capture severe damage resulting from significant early injury, as damage induced by SAH is known to occur early and progress over a prolonged period of time [33]. The analysis of groups beyond the 30-day point, such as 60 or 90 days, may provide additional insights into the evolution of regional injury and the potential for spontaneous recovery. Furthermore, while many neurobehavioral deficits could be detected via multiple assessments, some deficits are challenging or impossible to assess in combination. For example, the sucrose preference test of depression-like behavior requires food and water deprivation [70], which will invalidate other assessments done in the same period.

## CONCLUSION

Patients who survive SAH often experience disabling impairments, and currently available clinical techniques have limited ability to detect damage before it becomes irreversible. In this study, we have shown that a comprehensive battery of behavioral assessments is a highly accurate early warning for damage affecting specific cerebral regions. Specifically, behavioral tests of sensorimotor function, memory, anxiety-like and depression-like behaviors were found to predict damage in the cortex and basal ganglia, hippocampus, amygdala, and thalamus, respectively. Our findings suggest that these behavioral assessments can detect regional damage with acceptable accuracy, sensitivity, and specificity, with the notable exception of the hypothalamus. It is possible that combinations of behavioral assessments could further augment the diagnostic sensitivity or specificity of the assessments used here. Additional research into cell-specific damage and the evolution of SAH-induced injury may better characterize the threshold of behavioral assessments required to detect damage. Armed with this knowledge, clinicians may be able to intervene with targeted therapies to prevent permanent damage and improve long-term outcomes.

## Data Availability

The authors declare that all supporting data are available within this article.
